# SARS-CoV-2 Humoral and Cellular Immune Responses in People Living with HIV

**DOI:** 10.3390/vaccines12060663

**Published:** 2024-06-16

**Authors:** Simona Ruta, Corneliu Petru Popescu, Lilia Matei, Camelia Grancea, Adrian Marius Paun, Cristiana Oprea, Camelia Sultana

**Affiliations:** 1 Faculty of Medicine, Carol Davila University of Medicine and Pharmacy, 050474 Bucharest, Romania; simona.ruta@umfcd.ro (S.R.); cristiana.oprea@spitalulbabes.ro (C.O.); madalina.sultana@umfcd.ro (C.S.); 2 Stefan S. Nicolau Institute of Virology, 030304 Bucharest, Romania; liliamatei@yahoo.com (L.M.); cgrancea@yahoo.co.uk (C.G.); 3 Dr. Victor Babes Hospital of Infectious and Tropical Diseases, 030303 Bucharest, Romania; adrianpaun96@gmail.com

**Keywords:** COVID-19, SARS-CoV-2, vaccination, cellular, humoral immune response, immunosuppression

## Abstract

Immunosuppressed individuals, such as people living with HIV (PLWH), remain vulnerable to severe COVID-19. We analyzed the persistence of specific SARS-CoV-2 humoral and cellular immune responses in a retrospective, cross-sectional study in PLWH on antiretroviral therapy. Among 104 participants, 70.2% had anti-S IgG antibodies, and 55.8% had significant neutralizing activity against the Omicron variant in a surrogate virus neutralization test. Only 38.5% were vaccinated (8.76 ± 4.1 months prior), all displaying anti-S IgG, 75% with neutralizing antibodies and anti-S IgA. Overall, 29.8% of PLWH had no SARS-CoV-2 serologic markers; they displayed significantly lower CD4 counts and higher HIV viral load. Severe immunosuppression (present in 12.5% of participants) was linked to lower levels of detectable anti-S IgG (*p* = 0.0003), anti-S IgA (*p* < 0.0001) and lack of neutralizing activity against the Omicron variant (*p* < 0.0001). T-cell responses were present in 86.7% of tested participants, even in those lacking serological markers. In PLWH without severe immunosuppression, neutralizing antibodies and T-cell responses persisted for up to 9 months post-infection or vaccination. Advanced immunosuppression led to diminished humoral immune responses but retained specific cellular immunity.

## 1. Introduction

Clinical trials and real-life studies have extensively evaluated the SARS-CoV-2 immune responses both after natural infection and after vaccination, demonstrating preserved protection against hospitalization and deaths, with diminishing efficacy against symptomatic infections caused by the continuously evolving, immune-evasive viral variants [1]. Nevertheless, there are several vulnerable populations who are still at risk for severe forms of COVID-19, including immunosuppressed persons. An analysis of the data gathered in the WHO Global Clinical Platform on COVID-19 revealed an independent correlation between HIV infection and severe outcomes of patients hospitalized with SARS-CoV-2 infection [2]. Consequently, the European AIDS Clinical Society strongly recommends vaccination of people living with HIV (PLWH), irrespective of CD4 count and HIV viral load. Variant adapted booster doses are also recommended with high priority for PLWH [3,4].

Passive immunization using anti-SARS-CoV-2 monoclonal antibodies, used as a possible pre-exposure prophylaxis in PLWH (especially in those unvaccinated or with advanced immunosuppression), is no longer used due to the diminishing efficacy against the currently circulating SARS-CoV-2 variants [3].

In PLWH with controlled HIV infection, who are effectively treated, immunocompetent, and with suppressed viral replication, SARS-CoV-2 infection has similar incidence rates and comparable evolution with the general population [5]. Nevertheless, PLWH with advanced HIV disease and severe immunodeficiency are prone to a more severe evolution of COVID-19, with a higher risk of hospitalization and increased mortality [6,7]. After the emergence of the Omicron variant and its continuous antigenic drift, numerous breakthrough SARS-CoV-2 infections have been reported in all PLWH, including in vaccinated persons, with a higher frequency compared to the general population [8]. In addition, persistent SARS-CoV-2 infections, with continuous viral replication and high variability, have been reported in immune-suppressed persons infected with HIV, representing a potential source of new SARS-CoV-2 viral variants [1,9]. Repeated breakthrough infections could also potentially increase the HIV reservoirs in PLWH, even in those with undetectable HIV RNA in plasma on combination antiretroviral therapy (cART) [10].

Data on anti-SARS-CoV-2 immune response efficiency in PLWH are controversial. While some studies indicate similar humoral and cellular immune responses to those developed in HIV-negative subjects [11,12,13,14,15], others suggest a decreased immune response efficiency in PLWH [16,17,18]. Severe immunosuppression decreases the ability to mount specific immune responses, both after infection and vaccination [5,19], and more severe breakthrough infections were diagnosed in PLWH even during the early COVID-19 waves [7,20]. As such, data regarding the SARS-CoV-2 immunity in patients infected with HIV are still needed and are continuously gathered across diverse global regions with variable HIV demographics and healthcare standards [6].

The objective of the present study was to assess the seroprevalence of SARS-CoV-2 infection in non-hospitalized PLWH under antiretroviral therapy and to analyze the persistence of specific SARS-CoV-2 humoral and cellular immune responses after vaccination and/or infection.

## 2. Materials and Methods

### 2.1. Patients and Samples

This retrospective, cross-sectional study included 104 patients infected with HIV who were in active care in a regional HIV center in Victor Babes Hospital for Tropical and Infectious Diseases, Bucharest, Romania, between April and August 2022. The study was conducted in accordance with the Declaration of Helsinki and approved by the Hospital’s Ethics Committee; all participants have signed an informed consent. Ten mL of venous blood on EDTAK3 anticoagulant were collected from all the patients. Serologic and virologic determinations were carried out at Stefan S. Nicolau Institute of Virology, Bucharest, Romania. Plasma was centrifugated and stored at −20 °C. Peripheral blood mononuclear cells (PBMCs) were isolated using Ficoll-Paque PREMIUM (GE Healthcare, Stockholm, Sweden), washed twice with phosphate saline buffer (PBS), and cryopreserved at −80 °C in AIM-V Medium (Thermo Fisher Scientific, Waltham, MA, USA) with 10% DMSO, for further functional studies.

### 2.2. Detection of SARS-CoV-2 Humoral Immune Response

#### 2.2.1. SARS-CoV-2 Anti-Spike (S) IgG and IgA, and Anti-Nucleocapsid (NCP) IgG Immunoassays

Anti-S IgG antibodies were tested using a quantitative immunoenzymatic assay (EUROIMMUN Medizinische Labordiagnostika AG, Lübeck, Germany), with results expressed in RU/mL, and transformed in BAU/mL using a conversion factor of 3.2 [21]. The results were considered negative if <8 RU/mL (<25.6 BAU/mL); borderline if between 8–11 RU/mL (25.6–35.2 BAU/mL); positive > 11 RU/mL (>35.2 BAU/mL).

Anti-S IgA antibodies and anti-NCP IgG antibodies were tested with commercially semiquantitative enzyme-linked immunosorbent assays (EUROIMMUN Medizinische Labordiagnostika AG, Lübeck, Germany) according to the manufacturer protocols. Optical density (OD) was assessed at 450 nm, and the results were expressed as reactivity (a ratio calculated as the OD 450 of the patient’s sample over the OD 450 of a calibrator—an anti-S IgA positive sample or anti-NCP IgG positive sample provided by the manufacturer). The resulting ratio was considered negative if <0.8, borderline if ≥0.8 to <1.0, and positive if ≥1.1.

#### 2.2.2. Surrogate SARS-CoV-2 Virus Neutralization Test

A surrogate virus neutralization test (GenScript cPass™ SARS-CoV-2 Neutralization Antibody Detection Kit, Genscript, Piscataway, NJ, USA) was used to test the presence of neutralizing antibodies. This functional assay mimics the antibodies’ ability to inhibit the interaction between the ACE2 receptor and the spike receptor-binding domain (RBD) using the validated procedure [22]. Briefly, serially diluted samples are mixed with enzyme-conjugated RBD specific for the BA.4/BA.5 Omicron strain (the predominant variant circulating worldwide in 2022), incubated at 37 °C for 30 min, and after that interacted with recombinant human ACE2. After the incubation of 15 min, the plate was washed, and 100 μL tetramethylbenzidine was added for 15 min at RT. The samples’ neutralizing capacity was calculated using the formula: % inhibition = [1 − (OD450 of the Sample/Mean OD450 of Negative Controls] × 100 (OD450 = Optical density measured at 450 nm).

An inhibition value > 30% is positive for the SARS-CoV-2 neutralizing activity; values of 30–60% represent a low neutralizing activity, 60–90%—medium, and >90% indicating a high SARS-CoV-2 neutralizing activity.

### 2.3. Detection of Cellular Immune Response

#### 2.3.1. ELISpot Test

The IFN-γ and IL-2 secreting PBMCs were detected by an ELISpot assay (human IFN-gamma/IL-2 Dual-Color ELISpot Kit, R&D Systems, Santa Clara, CA, USA) using the manufacturer indications; cryopreserved PBMCs were thawed and let to rest in AIM-V Medium (Thermo Fisher Scientific, USA) before plating—two hours, 37 °C. The cells were plated at 2 × 10^5^ cells/well and were stimulated for 20 h with 2 µg/mL of the recombinant BA.4/BA.5 S1 protein (R&D Systems, USA). Positive controls consisted of PBMCs stimulated with 2 µg/mL PepTivator^®^ CMV pp65 (Miltenyi Biotec, Gaithersburg, MD, USA)—a pool of 15-mer peptides with 11–amino acid overlap that covers the complete sequence of pp65 protein of cytomegalovirus (CMV), and negative control consisted of unstimulated PBMCs. AID ELISpot Reader System (Autoimmun Diagnostika GmbH, Straßberg, Germany) was used for spot count. All assays were performed in duplicate. The difference in spot-forming units per 10^6^ PBMCs between the spike peptide-stimulated PBMCs and negative controls represents the final result (ΔSFU/10^6^ PBMCs); negative values were set to zero.

#### 2.3.2. Flow Cytometry Analysis

The CD4/CD8 ratio was evaluated by flow cytometry using tetraCHROME CD45-FITC/CD4-PE/CD8-ECD/CD3-PC5 antibody Cocktail (Beckman Coulter, Brea, CA, USA) according to the manufacturer protocol. EPICS XL flow cytometer (Beckman Coulter, USA) and Kaluza software version 1.3 (Beckman Coulter, USA) were used to acquire the results.

### 2.4. Statistical Analysis

GraphPad Prism version 8 software was used for statistical analysis. Two-way analysis of variance (ANOVA), followed by a Tukey multiple comparisons test, was used for multiple groups comparation. For correlation analysis, the non-parametric Spearman test was utilized. The significance threshold was defined by *p* < 0.05.

## 3. Results

### 3.1. General Characteristics of the Enrolled Subjects

The study included 104 PLWH (65.4% males, mean age 38.4 ± 0.98 years, mean duration of HIV infection 127.3 ± 10.55 months). HIV infection was acquired by sexual transmission (58.7% of cases, either hetero or homosexual), parenteral transmission (32.7% by injecting drug use, 4.8% parenterally infected during early childhood), and by vertical transmission (3.8% of cases). The mean CD4 count at the time of the study was 606.6 ± 32.62 cells/mm^3^, 27.9% of the patients had mild immunosuppression (CD4 200–500 cells/mm^3^), and 12.5% had severe immunosuppression (CD4 < 200 cells/mm^3^). The general characteristics of the study patients divided by the degree of immunosuppression are presented in Table 1.

All patients were treated with cART at the time of the study, with a mean length of antiretroviral therapy of 120.5 ± 9.9 months. All regimens included a backbone of 2 nucleoside analogs reverse transcriptase inhibitors (NRTI), combined with an integrase inhibitor (INSTI) in 74% of cases, a protease inhibitor (PI) in 18.3% of cases, and a non-nucleoside reverse transcriptase inhibitor (NNRTI) in 7.7% of cases. Only 60.6% of PLWH included in the study (63/104) had undetectable viral load, with significant differences according to the degree of immunosuppression, as shown in Table 1. Patients who were severely immunosuppressed have been diagnosed more recently and had lower CD4 nadir counts and higher HIV viral loads.

Out of 104 PLWH, only 40 (38.5%) were vaccinated against SARS-CoV-2 infection, either with an mRNA vaccine (Pfizer-BioNTech vaccine, BNT162b2—16 patients), or with a viral-vector based vaccine (Astra-Zeneca vaccine, ChAdOx1-S—11 patients, or Janssen, Ad26.COV2.S—13 patients). Just eight patients had received a booster dose; all boosters were monovalent mRNA vaccines with the ancestral SARS-CoV-2 strain. The mean duration of time between the last vaccine dose and enrollment was 8.76 ± 4.1 months.

Only 11 subjects out of 104 tested PLWH (10.6%) had been previously diagnosed with a SARS-CoV-2 infection, all with a mild clinical form. The mean time length between infection and enrollment was 9.36 ± 1.6 months.

### 3.2. SARS-CoV-2 Seroprevalence

A total of 73 patients (70.2%) had anti-Spike IgG antibodies (mean titer 477.3 ± 232 BAU/mL), and 58 (55.8%) also had neutralizing antibodies against the Omicron variant (an inhibition value >30% in the surrogate SARS-CoV-2 virus neutralization test). Only 24 patients (23%) had anti-NCP antibodies (mean reactivity 2.3 ± 0.7) associated with anti-S IgG antibodies, indicating a prior SARS-CoV-2 infection.

Anti-S IgA antibodies were present in 62 patients (59.6%, with a mean reactivity of 4.2 ± 1.1), always associated with anti-S IgG antibodies.

All vaccinated patients had anti-S IgG antibodies, and 12 of them also had anti-NCP antibodies, signaling a prior SARS-CoV-2 infection and, thus, hybrid immunity. Of 40 vaccinated patients, 30 (75%) had detectable neutralizing antibodies against the Omicron variant (neutralizing activity in the surrogate SARS-CoV-2 virus neutralization test higher than 30%) and anti-S IgA antibodies.

The serologic profile of the previously SARS-CoV-2-infected patients was more heterogeneous. Interestingly, 4 out of the 11 patients who declared past COVID-19 had no SARS-CoV-2 antibodies (negative for anti-S IgG and IgA, and for anti-NCP IgG), and 2 patients had only anti-S IgG antibodies (mean titer 636.2 ± 124 BAU/mL), with no detectable anti-NCP antibodies. Nevertheless, the other 33 patients without a prior history of SARS-CoV-2 infection or vaccination displayed anti-S IgG antibodies (with or without anti-NCP antibodies), indicating asymptomatic, undiagnosed infections.

**Figure 1:**
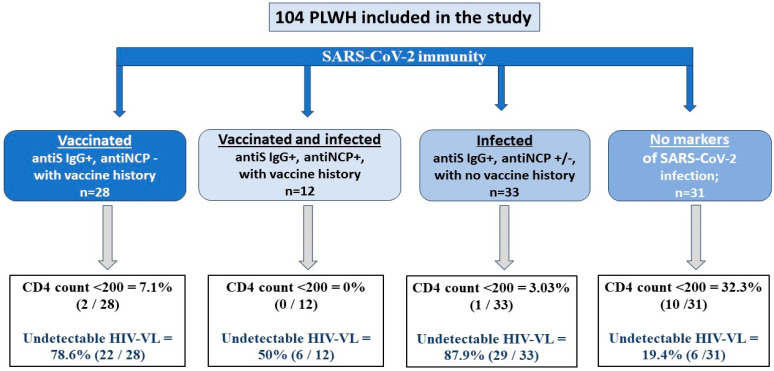
Patients’ distribution by SARS-CoV-2 serologic status and vaccination/infection history. The classification was based on the presence or absence of SARS-CoV-2 serological markers and self-declared vaccine history.

The patients’ distribution according to their SARS-CoV-2 serologic status and vaccination/infection history is shown in Figure 1.

As can be seen in Figure 1, out of all enrolled patients, 31 have no SARS-CoV-2 serologic markers; they have significantly decreased CD4 counts (mean 443.8 ± 64.49 vs. 670.4 ± 35.3; *p* = 0.0015) and significantly lower percentages of undetectable HIV viral load (19.4% vs. 78.1%; *p* = 0.021) compared to those with detectable SARS-CoV-2 antibodies.

### 3.3. Antibody Response According to SARS-CoV-2 Vaccination/Infection Status

**Figure 2:**
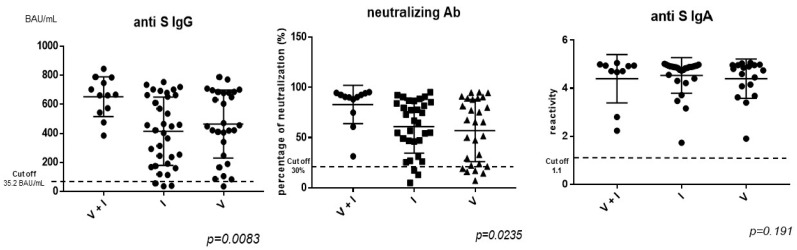
Antibody responses according to SARS-CoV-2 infection/vaccination status. V = SARS-CoV-2 vaccinated; I = SARS-CoV-2 infected; V + I = SARS-CoV-2 vaccinated and infected (with hybrid immunity).

There is no significant difference between PLWH who were vaccinated and those who were infected in terms of anti-S IgG titers or neutralizing capacity. However, PLWH with hybrid immunity against SARS-CoV-2 (infection and vaccination, with preserved serologic markers according to their serologic status—anti-S IgG and anti-NCP, associated with a history of vaccination) have significantly higher levels of anti-S IgG antibodies (mean titer 653.3 BAU/mL vs. 412.4 BAU/mL in those infected only, and 464.3 BAU/mL in those vaccinated only), and higher neutralization capacity (82.96% vs. 60.97% in those infected only, and 57.06% in those vaccinated only), as observed in Figure 2. There is a significant correlation between the anti-S IgG titers and the neutralizing activity against the Omicron variant. There are no significant differences in the antibody titers according to the time since vaccination (vaccinated <6 months vs. vaccinated 6–12 months ago vs. vaccinated >12 months). The reactivity of anti-spike IgA antibodies is similar in all patients (vaccinated, infected, or with hybrid immunity).

### 3.4. SARS-CoV-2 Humoral Immunity in Severe Immunosuppressed PLWH

As shown in Table 2, severe immunosuppression (CD4 count < 200 cells/mm^3^) is associated with significantly lower percentages of patients with detectable anti-S IgG (*p* = 0.0003), anti-S IgA antibodies (*p* < 0.0001) and with lack of neutralizing activity against Omicron variant (*p* < 0.0001). Patients with advanced immunosuppression have lower anti-S IgG antibody mean titers (*p* = 0.005) and anti-S IgA antibody reactivities (*p* = 0.002) compared to patients with mild or no immunosuppression. 

**Figure 3:**
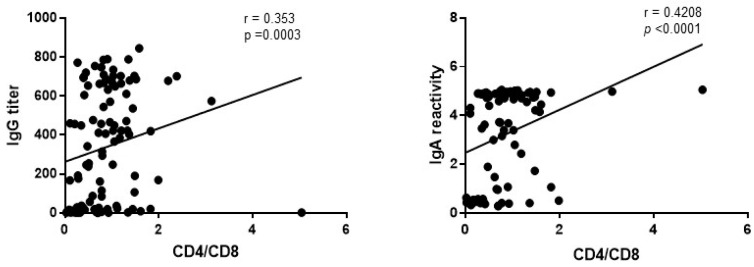
Correlation between anti-S IgG and CD4/CD8 (3A) and between anti-S IgA and CD4/CD8 (3B).

Moreover, there is a significant moderate correlation between the CD4/CD8 ratio and the titers of anti-S IgG and IgA antibodies (r = 0.353; *p* = 0.0003 and r = 0.4208; *p* < 0.0001, respectively, using non-parametric Spearman test)—Figure 3.

Patients with high HIV viral loads (>10^4^ copies per mL) display significantly lower levels of CD4 counts (330.8 ± 55.24 vs. 681.5 ± 34.34 cells/mm^3^) and decreased levels of SARS CoV-2 serological markers (mean anti-S IgG = 176.5 ± 267.4 vs. 369.4 ± 281.1 BAU/mL, mean anti-S IgA = 1.92 ± 0.4 vs. 3.6 ± 0.2, mean neutralizing Ab percentages = 62.8 ± 12.2 vs. 64.1 ± 3.4), as compared to those with lower or undetectable HIV viral loads.

### 3.5. SARS-CoV-2 Cellular Immunity

**Figure 4:**
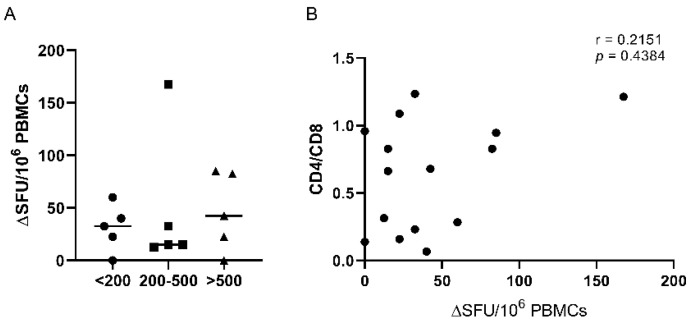
SARS-CoV-2-specific T-cell responses in HIV-positive subjects. (**A**). Number of IFN-γ SFU per 10^6^ PBMCs by CD4 counts following stimulation with recombinant SARS-CoV-2 BA.4/BA.5 S1 protein. (**B**). Correlation between CD4/CD8 ratio in individuals infected with HIV with their total SARS-CoV-2 responses. The non-parametric Spearman test was used for correlation analysis.

Cellular immunity was assessed in 15 PLWH similarly distributed in three groups: without immunosuppression—CD4 count > 500 cells/mm^3^, mild immunosuppression—CD4 count 200–500 cells/mm^3^ and severe immunosuppression—CD4 count < 200 cells/mm^3^. PBMCs from patients with mild and severe immunosuppression have inferior responses when stimulated with a recombinant Omicron BA.4/BA.5 S1 protein, as compared to patients without immunosuppression (Figure 4A).

Detectable IFN-γ producing T cell responses were present in 13 out of 15 evaluated patients (86.7%), including two patients with CD4 < 200 cells/mm^3^ who did not display any serological markers of SARS-CoV-2 infection. There were no detectable IL-2-secreting T cells. Subjects without immunosuppression (>500 CD4 cells/mm^3^) respond better to re-stimulation, although the cellular immune response displays individual heterogeneity. The non-parametric Spearman test did not detect any correlation between anti-SARS-CoV-2-specific cellular response and the CD4/CD8 ratio (r = 0.2151; *p* = 0.4384) (Figure 4B).

## 4. Discussion

We report that the majority of PLWH have preserved SARS-CoV-2 humoral and cellular immunity post-vaccination and post-infection, including anti-S IgA antibodies, neutralizing antibodies against the Omicron variant, and IFN-γ-secreting T cell responses. Moreover, we show that an important proportion of PLWH who have not been diagnosed with a prior SARS-CoV-2 infection and have not been SARS-CoV-2-vaccinated display serologic markers of SARS-CoV-2 infection, suggesting rather frequent asymptomatic infections, with preserved subsequent immunity.

Still, our results show important differences according to the degree of immunosuppression, with lower percentages of SARS-CoV-2 seropositive patients and lower SARS CoV-2 antibodies titers in PLWH who are severely immunocompromised (CD4 count < 200 cells/mm^3^), irrespective of their vaccination/infection status. A lower immunogenicity of SARS-CoV-2 vaccines has been demonstrated in numerous studies for PLWH who are severely immunocompromised, regardless of their HIV viral load [23,24,25,26]. On the contrary, PLWH on cART, with undetectable plasma HIV viral loads (less than 50 copies/mL) and CD4 counts of more than 350 cells/mm^3^, develop strong anti-SARS-CoV-2 humoral immune responses after vaccination, comparable in magnitude and persistence with those of people without HIV [12,23,24]. In our study, PLWH with severe immunosuppression (CD4 count < 200 cells/mm^3^) have significantly higher HIV viral loads; these are the ones with significantly decreased levels of SARS CoV-2 serological markers. Recent studies have suggested that viral suppression is an important factor for the durability of SARS-CoV-2 immunity, as PLWH with undetectable viral loads have similar immune responses with individuals from the general population after a third dose of SARS-CoV-2 mRNA vaccine and do not need supplementary booster doses. Still, the optimal timing and composition of future booster vaccinations in PLWH must be studied in association with the waning immunity, the emerging of new SARS-CoV-2 variants of concern, and the possibility of antigenic imprinting [27].

Current studies have also shown lower neutralization activity against all Omicron SARS-CoV-2 variants (SARS-CoV-2 B.1, B.1.617, BA.1, and BA.2) in PLWH with severe immunosuppression [28]. Nevertheless, the sub-variant specific neutralizing antibodies increase after a booster dose, reaching high levels in PLWH with a high CD4 nadir and lower levels in PLWH with a low CD4 nadir; no substantial difference was detected in the rate of specific antibodies drop several months after variant specific booster vaccination in PLWH and HIV negative individuals [29,30].

A third of the PLWH enrolled in this study displayed no serologic markers for SARS-CoV-2; all had significantly decreased CD4 levels and tended to have detectable viral load under antiretroviral treatment. This might indicate either an (improbable) lack of exposure to SARS-CoV-2 infection or previous asymptomatic infections, with a weaker immune response stimulation, followed by a complete waning of serologic markers. Data on decreased and volatile SARS-CoV-2 immune responses post-infection and post-vaccination have also been reported for patients with other causes of primary or secondary immunodeficiency (solid organ or stem cell transplantation recipients, patients on chimeric antigen receptor T cell therapy, or patients with chronic leukemias) [31,32,33]. The magnitude of the immune response in PLWH can be further influenced by the infecting viral variant, as some reports have demonstrated a higher neutralization activity against the Delta SARS-CoV-2 variant but a very decreased one for the Beta variant [34]. We did not find any correlation between the presence and magnitude of the SARS-CoV-2-specific immune responses in PLHW and their age, gender, or body mass index, although such associations were previously reported in the general population [35], probably due to the fact that most persons infected with HIV included in this study are young, without associated comorbidities.

Detectable SARS-CoV-2-specific cellular immune responses following re-stimulation with a BA.4/BA.5 Spike recombinant protein were present in almost all tested patients, although the sample size is rather small. The cellular immune responses were higher in PLWH without immunosuppression (CD4 > 500 cells/mm^3^). Nevertheless, SARS-CoV-2-specific cellular immune responses were also detected in two immunosuppressed PLWH, who had no serological markers of SARS-CoV-2 infection. This suggests a durable presence of cellular immunity irrespective of the individual’s level of immunosuppression. Earlier studies have already demonstrated that PLWH, virally suppressed and on ART, presents a robust T cell response able to cross-recognize SARS-CoV-2 variants [12,17,36,37] even when the humoral immune response showed low or absent neutralization of SARS-CoV-2 [17].

Still, in PLWH with CD4 < 200 cells/mm^3^, the cellular immune response induced by mRNA vaccines was inferior to the one developed in PLWH without immunosuppression or in healthy controls [24]. Follow-up studies on the persistence of the cellular immune responses in PLWH showed a decline 6 months after vaccination, but with no significant difference compared to healthy controls. In immunocompetent individuals, studies on the long-term durability of the cellular immune responses against SARS-CoV-2 suggest that memory T-cell responses initiated by the first viral infection retain significant cross-reactivity even after a span of 2 years [38,39].

In our study, PLWH with hybrid immunity (acquired after vaccination and infection) display the strongest immune responses against SARS-CoV-2, similar to the data available from immunocompetent persons [40,41]. This is in accordance with previous reports, showing a significant increase in antibody titers and neutralizing capacity after breakthrough SARS-CoV-2 infections in PLWH [42]. Noteworthy, in the present study, SARS-CoV-2 anti-S IgA antibodies, a surrogate marker for local immunity, were still present in most PLWH at 8–9 months after infection or vaccination, similar to the data reported in longitudinal follow-up studies in the general population, showing persistent adaptative systemic and local immune responses up to two years, with more durable and organized immune responses after recovery from COVID-19 [38]. Recent studies have also demonstrated that breakthrough infections with the currently circulating Omicron variants enhance both the breadth and persistence of all types of specific immune responses, including nasal spike-specific IgA levels [43]. Longitudinal studies have also demonstrated a rapid activation of SARS-CoV-2-specific cellular immune responses during breakthrough infections, explained both by the recall of CD4+ and CD8+ spike-specific memory cells and de novo T cell responses [44]. Increased anti-SARS-CoV-2 IgA titers have been associated with better viral neutralization and protection against breakthrough infections [45,46]. Taken together, these results support the prioritization of PLWH with severe immunosuppression for SARS-CoV-2 vaccination and variant-adapted booster administration. Although controversy regarding the ability of intramuscular vaccination to elicit IgA antibodies persists [18,47,48], our results support the data showing that vaccinated persons have durable systemic IgA responses, correlated with the local mucosal immunity, adding a supplementary layer of protection against SARS-CoV-2 infection [49]. Individual variability might be related to the heterogeneity of the oral and/or gut microbiome composition [50,51], and modulated by subsequent breakthrough infections [52]. Intranasally administered vaccines can help boost local protection and decrease SARS-CoV-2 transmission [53,54].

A meta-analysis including 22 studies and 6522 PLWH reported that a second dose of the SARS-CoV-2 vaccine is associated with improved seroconversion, although with lower titers of antibodies in PLWH than in immunocompetent subjects [55]. Another meta-analysis based on 50 studies reported increases in the seroconversion rate achieved by PLWH after each new dose of vaccine; that remains, however, inferior to the one acquired in the general population [56]. In our study, only eight individuals received a vaccine booster dose; this low percentage reflects the general low level of booster uptake in Romania (<10%) of the general population) [57]. Administration of additional vaccine doses (up to two variant-adapted boosters) in PLWH has been associated with an expansion of the specific humoral and cellular immune responses that are maintained for up to 6 months [14]. Moreover, even in PLWH with CD4 < 200 cells/mm^3^, there is an increase in the humoral response after a third dose of the SARS-CoV-2 vaccine, especially in the case of heterologous vaccination, while the cellular immune response appears to remain more stable [58]. Our results show that PLWH with preserved cellular immunity (>500 CD4 cells/mm^3^) display improved cellular immune responses, with higher numbers of IFN-γ secreting T cells after stimulation with a SARS-CoV-2 Omicron S1 protein. The absence of IL-2-secreting cells might be explained by the mild or asymptomatic SARS-CoV-2 infections in our study participants. Previous reports have signaled that IL-2-secreting cells are less frequent in those with mild disease compared to those with severe or moderate forms of COVID-19 [59]. In addition, in vaccinated PLWH, SARS-CoV-2-specific T cells predominantly produce IFN-γ and TNF-α, while cells producing IL-2 are very rare [60]. The main limitation of this study is the relatively small number of participants who have been evaluated for the cellular immune response. Nevertheless, the data are still valuable, as they show frequent asymptomatic SARS-CoV-2 infection in individuals infected with HIV, with subsequent persistence of cellular immunity, even in persons without any serological markers of SARS-CoV-2 infection. Moreover, we add data on the persistence of neutralizing antibodies able to recognize the Omicron variant in patients who were vaccinated with only two doses of the initial vaccine, of interest for future vaccination strategies for PLWH, potentially adapted according to their immunosuppression status.

## 5. Conclusions

SARS-CoV-2-specific immune response in PLWH without immunosuppression is preserved, with neutralizing antibodies and T-cell responses persisting for up to 9 months after infection and/or vaccination. Similar to immunocompetent individuals, hybrid immunity (SARS-CoV-2 infection plus vaccination) is associated with stronger and more persistent immune responses. Noteworthy, in severely immunosuppressed persons who maintain high HIV viral loads, waning of all SARS coV-2 serologic markers is frequent. Longitudinal studies are needed to investigate the long-term durability of the SARS-CoV-2 immune response in the presence of multiple circulating viral variants.

## Figures and Tables

**Table 1:** Characteristics of the study PLWH with or without immunosuppression.

Patients’ Immunological Status	CD4 Count > 500 n = 62	CD4 Count 200–500 n = 29	CD4 Count < 200 n = 13	*p*
Male, n (%)	41 (66.1%)	20 (68.9%)	9 (69.2%)	0.9009
Age (years) mean ± SD	38.3 ± 9.7	38.8 ± 12.3	38.3 ± 4.6	0.9736
AIDS C3 (%)	27.4	34.5	35	0.7107
HIV infection duration (months), median [IQR]	132 [56–240]	78.5 [23.5–144]	72.5 [1–122]	**0.0376**
Age at HIV diagnosis (years), median [IQR]	27 [14–35]	26 [21–36]	33.5 [24–37.5]	0.5185
cART treatment duration (month), mean ± SD	108 [60–228]	72 [36–204]	60 [2–120]	0.1343
CD4 nadir (cells/mm^3^), mean ± SD	328.9 ± 267.4	175.2 ± 120.5	86.8 ± 71.9	**0.0003**
CD4/CD8 ratio, mean ± SD	1.16 ± 0.7	0.63 ± 0.3	0.13 ± 0.08	**<0.0001**
HIV viral load (log10 HIV RNA copies/mL), mean ± SD	4.23 ± 3.9	5.1 ± 4.5	5.38 ± 4.6	0.005
Zenith HIV viral load (log10 copies/mL), mean ± SD	5.65 ± 4.8	5.72 ± 4.9	6.1 ± 5.7	0.1023
Undetectable HIV RNA, n (%)	45 (72.6%)	16 (55.2%)	2 (15.4%)	**0.0005**
HIV RNA > 4 log10 copies/mL, n (%)	6 (9.7%)	9 (31.03%)	8 (61.2%)	**<0.0001**
Number of cART regimens, mean ± SD	2.9 ± 2.3	2.96 ± 2.6	2.3 ± 1.6	0.6708

SD = Standard deviation; AIDS C3 = HIV clinical stage C3, defined by clinical conditions indicative of severe immunosuppression or CD4 count <200 cells/mm^3^; IQR = Interquartile range; cART = combination antiretroviral therapy.

**Table 2:** Anti SARS-CoV-2 serological status according to the immunosuppression level.

Patients’ Immunological Status	CD4 Count > 500 n = 62	CD4 Count 200-500; n = 29	CD4 Count < 200 n = 13	*p*
Positive for anti-S IgG antibodies, n (%)	49 (79.1%)	21 (72.4%)	3 (23.1%)	**0.0003**
Anti-S IgG titer (BAU/mL) mean ± SD	377.6 ± 276.1	354.5 ± 301.1	106.8 ± 228.1	**0.005**
Positive for anti-S IgA antibodies n (%)	56 (90.3%)	21 (72.4%)	4 (30.8%)	**<0.0001**
Anti-S IgA antibodies reactivity, mean ± SD	3.6 ± 1.7	3.2 ± 1.9	1.4 ± 1.1	**0.002**
Positive for neutralizing activity (>30%), n (%)	51 (82.3%)	23 (79.3%)	2 (15.4%)	**<0.0001**
SARS-CoV-2 neutralizing capacity, mean ± SD	63.8 ± 27.6	64.2 ± 30.1	45.5 ± 29.7	**0.041**

SD = Standard deviation.

## Data Availability

Data is unavailable due to privacy or ethical restrictions.
